# Increased Activity of Cell Membrane-Associated Prothrombinase, Fibrinogen-Like Protein 2, in Peripheral Blood Mononuclear Cells of B-Cell Lymphoma Patients

**DOI:** 10.1371/journal.pone.0109648

**Published:** 2014-10-10

**Authors:** Esther Rabizadeh, Izhack Cherny, Ofir Wolach, Shany Sherman, Natalia Binkovski, Alon Peretz, Doron Lederfein, Aida Inbal

**Affiliations:** 1 Hemato-Oncology Laboratory, Felsenstein Medical Research Center, Rabin Medical Center, Petach Tikva, Israel, affiliated with Sackler Faculty of Medicine, Tel Aviv University, Tel Aviv, Israel; 2 Hematology Laboratory, Rabin Medical Center, Petach Tikva, Israel; 3 Thrombosis and Hemostasis Unit, Hematology Institute, Rabin Medical Center, Petach Tikva, Israel, affiliated with Sackler Faculty of Medicine, Tel Aviv University, Tel Aviv, Israel; 4 Occupational Health Clinic, Rabin Medical Center, Petach Tikva, Israel; University Medical Center of the Johannes Gutenberg University of Mainz, Germany

## Abstract

Fibrinogen-like protein 2, FGL-2, was reported to be overexpressed in various cancer tissues, where it acts as a transmembrane prothrombinase. This study aims to determine the prothrombinase activity of FGL-2 in peripheral blood mononuclear cells (PBMC) of patients with B-cell lymphoma. FGL-2 activity was determined in patients with B-cell lymphoma (n = 53), and healthy controls (n = 145). FGL-2 activity in patients at diagnosis increased 3±0.3 fold (p<0.001). Sensitivity and specificity of the test was established at 73.6% and 80.7%, respectively, using a cutoff of 150% activity over control. Moreover, FGL-2 activity in 10 of 11 patients in remission decreased by 76%. In contrast, no significant difference was observed in expression levels of fgl-2 gene in patients and controls. Taken together, our study indicates that FGL-2 prothrombinase activity in PBMC of lymphoma patients is increased in active disease and normalizes during remission, thus being a potential marker for follow up of lymphoma patients.

## Introduction

Hematologic and solid tumors are associated with hypercoagulability, the reason for which has not been delineated. Malignant cells are known to directly activate blood coagulation by producing procoagulant, fibrinolytic, and proaggregating factors, releasing proinflammatory and proangiogenic cytokines (such as TNF-α, interleukin-1ß); and interacting directly with host endothelial cells, leukocytes, and platelets, via adhesion molecules [1]. However, the precise procoagulant proteins that stimulate tumorigenesis are as yet unknown [1], [2], [3], [4], [5]. Blom et al observed the highest risk of venous thromboembolism (VTE) among patients with hematological malignancies (odds ratio 28), followed by lung cancer (odds ratio 22) and gastrointestinal cancer (odds ratio 20) [3]. Consistent with these findings is that deep vein thrombosis originating in the lower limbs was the most common form of thrombotic disease in adults with acute lymphatic leukemia (38.9%) [3], [6]. Lymphoma is the most common blood cancer. Non-Hodgkin's lymphoma (NHL) encompasses a heterogeneous group of malignancies, in which approximately 85–90% of lymphomas are derived from B lymphocytes. Wide variations exist in disease histology, biology and in the clinical approach to each lymphoma subtype [7]. The annual incidence of B-Cell lymphoma is estimated at 15–50 cases/100,000 [8] and it comprises the most common group of hematologic malignancies [9]. The staging and prognostic stratification of patients with lymphoma is generally based on clinical scores such as the International Prognostic Index (IPI) [10], [11], [12]. The response to therapy is also based mainly on clinical assessment tools such as positron emission tomography [13]. Thus, novel biomarkers that may assist in diagnosis and assessment of response to therapy are eagerly anticipated.

Among the suggested candidates is cell membrane-associated protein, fibrinogen-like protein 2 (FGL-2), a member of the fibrinogen family of proteins [14]. Previous studies have reported that FGL-2 was detected in tumor tissues, being overexpressed in the tumor and interstitial inflammatory cells [15]. However, the prothrombinase activity of FGL-2 in PBMC of cancer patients has not been studied yet.

FGL-2 (also known as FGL-2-prothrombinase) exerts serine protease activity and is capable of directly cleaving prothrombin to thrombin in the absence of factor VII or factor X. Like plasmatic prothrombinase - factor Xa, FGL-2 prothrombinase requires phospholipids, calcium, and factor Va for optimal catalytic activity [16]. However, unlike factor Xa, FGL-2 is a transmembrane protein which is not inhibited by antithrombin in the presence of heparin or by other protease inhibitors that inhibit factor Xa [16].

FGL-2 is expressed on the surface of activated macrophages and endothelial cells and also secreted by peripheral blood CD4+ and CD8+ T cells. The secreted protein is devoid of coagulation activity. It has potent modulatory effects on the adaptive immune system and was reported to inhibit the maturation of dendritic cells [17], [18], [19]. The prothrombinase and immune activities of FGL-2 are located on distinct domains on the FGL-2 molecule [17].

Recombinant FGL-2 protein was previously shown to induce sprouting in vascular endothelial cells [18]. The coagulant activity of FGL-2 was first described in a murine fulminant hepatitis model [19]. The prothrombinase activity of FGL-2 is exhibited when it is expressed in activated macrophages and endothelial cells in the form of a membrane-associated protein. FGL-2 has been shown to be associated with both experimental and human allograft rejection that was abrogated following neutralization of FGL-2 by antibodies or in FGL-2 knockout mice [20]. Macrophage and endothelial cell induction of FGL-2 occurs *via* interferon gamma (IFN –γ) [21]. Recently, it was reported that knock down of FGL-2 delayed tumor growth and angiogenesis in mice injected with human hepatocellular carcinoma cell line [22].

In the present work we measured FGL-2 prothrombinase activity and mRNA expression in peripheral blood mononuclear cells (PBMC) of patients with B-cell lymphoma.

## Materials and Methods

### Patients and controls

The study group consisted of 53 consecutive patients with indolent (n = 28) or aggressive (n = 25) lymphoma who were diagnosed at the Hematology Institute, Rabin Medical Center between November 2011 and July 2013 (Table 1). Blood samples were collected from each patient and from healthy controls (n = 145) for testing FGL-2 activity and mRNA.

**Table 1:** Patient characteristics.

Characteristics	Aggressive lymphoma (n = 25)	Indolent lymphoma (n = 28)	All patients (n = 53)
Age, median (range)	69 (29–83)	65 (39–85)	66 (29–85)
Female, n (%)	12 (48%)	11 (39%)	23 (44%)
Stage, n (%)^a^			
1	2 (8%)	2 (7%)	4 (8%)
2	5 (20%)	2 (7%)	7 (13%)
3	1 (4%)	8 (29%)	9 (17%)
4	17 (68%)	14 (50%)	31 (59%)
Extranodal disease	11 (44%)	8 (29%)	19 (36%)^b^
LDH (IU/L), median	447	388	413

a According to the Ann Arbor staging system.

b Data was not available for extra nodal status and B symptoms in 6 patients and 26 patients, respectively.

In addition, blood samples were collected from hospitalized individuals undergoing coronary bypass surgery, who served as a model for major tissue surgery-induced injury which requires extensive generation of thrombin (n = 11). The patient charts were reviewed and epidemiological, clinical and outcome data were collected. In order to alert a possible bias between the patient and the control groups, the age and gender of each human subject was documented. Moreover, the following patients' parameters were documented: stage of the disease, histology, lactate dehydrogenase (LDH) level, B symptoms, performance status (according to the Eastern Cooperative Oncology Group [ECOG] scale) and extra-nodal involvement. Patient outcomes as well as any anti neoplastic drugs administered during blood sample acquisition were also documented. Thirty two percent of lymphoma patients were tested while not being treated with anti-neoplastic drugs, 32% were tested while receiving chemotherapy and 14% received steroid prophase while being tested. No data regarding therapy were available for 11 tested lymphoma patients. In eleven B-Cell lymphoma patients blood samples for FGL-2 testing were obtained in complete remission. The study was approved by the Local Ethics Committee of the Hospital and informed consent was signed by each participant.

### Mononuclear Cell Isolation

PBMC were isolated from heparinized peripheral blood and placed on sterile plastic UNI-SEP tubes (Novamed #U-02, Jerusalem, Israel) containing a solution of 5.6% polysucrose and 9.6% sodium metrizoate (density 1.077 g/ml). The cells were centrifuged at 1000×g for 20 min at 18–20°C. PBMC were collected and washed 3 times with PBS.

### Fgl-2 Prothrombinase Activity in PBMC

FGL-2 activity was measured by thrombin generation assay, based on that published by Ghanekar *et*.*al*. [20], with slight modifications. A total of 30×10^6^ cells/ml (PBMC) were homogenized by three times freezing/thawing cycles. Thereafter, 50 µl of the cell extract was mixed with human prothrombin (Stago, Asnieres, France) in a reaction buffer (20 mM HEPES, 150 mM sodium chloride, 5 mM calcium chloride, pH 7.4) to a final concentration of 10 µM prothrombin. The reaction mixture was incubated for 30 min at 37°C, followed by the addition of 125 µl cold assay buffer (50 mM Tris, 227 mM sodium chloride, 1% bovine serum albumin and 1% sodium azide, pH 8.3). After centrifugation at 17760×g for 5 minutes, 140 µl of supernatant was transferred to a flat-bottom 96-well plate, and 16 µl of 0.6 µg/ml chromogenic substrate S-2238™ (H-D-Phenylalanyl-L-pipecolyl-L-arginine-p-nitroaniline, Chromogenix, Milano, Italy) were added to each well. Thrombin generation was measured at regular intervals at 405 nm using an automated plate reader. Thrombin activity was calculated in each sample by comparison with a standard curve generated using known concentrations of human thrombin (Omrix, Jerusalem, Israel). FGL-2 activity is reflected by the amount of thrombin generated and expressed as a percentage relative to mean FGL-2 activity of PBMC of healthy controls.

#### RT-PCR analysis of FGL-2

The amount of mRNA of FGL-2 was measured in PBMC by quantitative RT-PCR analysis. Total RNA was isolated using RNAqueous (Ambion #Am1912) and RT-PCR analysis was performed using Rotor-gene RG-3000 (Corbett). The difference in cycle time (Ct) was measured by comparing FGL-2 gene with ABL-1 gene (control). The relative copy number was calculated by the formula RQ = 2^−ΔCT^.

### FGL-2 ELISA

PBMC from lymphoma patients (n = 10) and normal controls (n = 9) were isolated and homogenized as specified above. Total protein concentration of each sample was determined using BCA Protein Assay Kit (Pierce, Rockford, USA). FGL-2 protein level was determined by ELISA using a specific FGL-2 LEGEND MAX ELISA kit (BioLegend, San Diego, CA, USA) according to manufacturer's instructions.

### Statistical Analysis

Statistical analyses were performed using SPSS ver.15.0. Square root transformation of the FGL-2 mean activity was carried out to approach normal distribution. The following statistical tests were employed: the student t-test was used to assess differences between the cohorts of control and patients FGL-2 mean values; Pearson's correlation was conducted to describe the association between FGL-2 activity and several variables such as age, LDH, gender, histology, extra-nodal involvement, presence or absence of B symptoms, outcome measures, stages of the disease and performance status of the patients; The Wilcoxon signed-rank test was used to assess differences in FGL-2 activity at diagnosis of lymphoma and in remission; Receiver operating curve (ROC) was generated to measure FGL-2 activity performance. A p-value of <0.05 was considered statistically significant.

## Results

The prothrombinase activity of FGL-2 was measured in PBMC by a thrombin generation assay of 53 patients with B-cell NHL (Table 1) and 145 healthy controls. The median age of the control group (38.5 years, range 19–78) was younger than that of the patients (66 years, range 29–85), however, age did not correlate with FGL-2 activity in either patient or control groups. The patients group included those with aggressive lymphoma (23 newly diagnosed or relapsed diffuse large B cell lymphoma [DLBCL], one Burkitt lymphoma and one post-transplant lymphoproliferative disease) as well as patients with indolent lymphoma (15 follicular lymphoma, 4 marginal zone lymphoma, 2 small lymphocytic lymphoma and 2 Mantel cell lymphoma). Five patients had indolent B cell lymphoma not otherwise specified. Patient characteristics are shown in Table 1.

**Figure 1:**
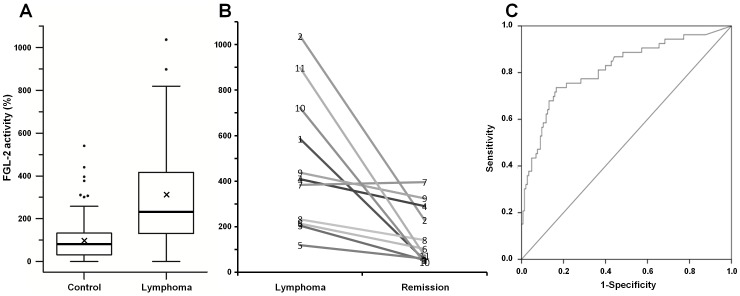
Prothrombinase activity of FGL-2 in PBMC. **A** - Boxplot presentation of FGL-2 activity in PBMC from lymphoma patients (n = 53) and controls (n = 145). FGL-2 activity was measured by thrombin generation assay and expressed as a percent activity relative to healthy controls. Thick line represents median activity and X indicates the mean activity. **B** - FGL-2 activity of eleven B-Cell lymphoma patients during active disease and remission. **C** - Sensitivity and specificity ROC analysis. The area under the curve is 0.817, standard error: 0.037.

Figure 1 illustrates FGL-2 prothrombinase activity in PBMC. FGL-2 activity of 53 patients was compared to that of 145 normal controls. A 3-fold increase in FGL-2 activity was observed in PBMC of patients compared to that of normal controls (mean ± SEM: 306%±% versus 100%±8%; p<0.001; median: 232% versus 83%; Figure 1A). There was no correlation between FGL-2 activity and age, gender, indolent versus aggressive histology, disease stage, extra-nodal disease, B-symptoms and no therapy versus steroids or chemotherapy. In addition, a correlation was not found between patient outcome and baseline FGL-2 activity levels.

Eleven lymphoma patients who reached complete remission following immuno-chemotherapy were also tested in remission (Figure 1B). In 10 out of the 11 patients FGL-2 activity decreased by a mean of 76% (ranging from 26% to 94%). In one patient, FGL-2 levels remained unchanged. There was no difference in clinical parameters or response to treatment between this patient and the rest of the group. Wilcoxon signed-rank test showed a significant post-therapy reduction in FGL-2 activity (p = 0.004).

To assess the strength of FGL-2 activity as a potential marker for active B-cell lymphoma, receiver operator curves were calculated (Figure 1C). Overall, the increase in FGL-2 activity over a cutoff value of 150% appeared to exhibit a sensitivity of 73.6% and specificity of 80.7% for the diagnosis of lymphoma.

To study if FGL-2 prothrombinase activity is affected by tissue damage induced by major surgery, which requires extensive generation of thrombin, we measured its activity in PBMC of patients undergoing coronary bypass surgery. The FGL-2 activity in PBMC of this cohort of patients was similar to that observed in healthy controls (Mean ± SEM, 99%±27.9%; Median, 94%; p-value versus healthy controls  = 0.97, p-value *versus* lymphoma patients <0.001).

**Figure 2:**
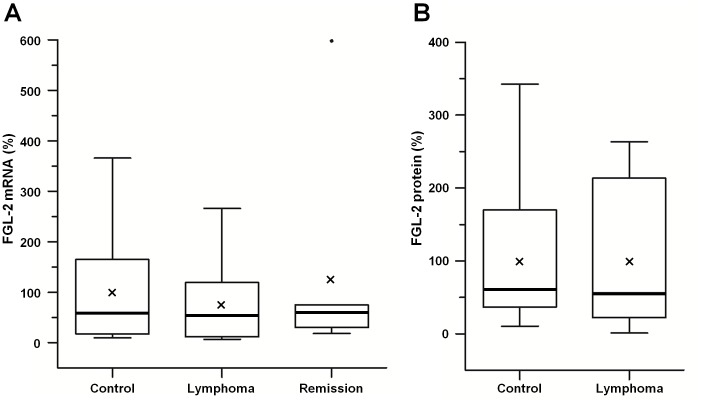
fgl-2 mRNA and protein levels analyses. Comparative boxplot presentation of fgl-2 mRNA and protein levels in PBMC of lymphoma patients and controls. **A**- Fgl-2 mRNA was analyzed by RT-PCR and determined relative to the house-keeping gene, abl-1. The mRNA levels of fgl-2 from PBMC of patients with active B-cell lymphoma (n = 12) and B-cell lymphoma in remission (n = 7) are expressed relative to healthy controls (n = 13). **B** - FGL-2 protein level was analyzed by ELISA. The protein levels of FGL-2 from PBMC of patients with active B-cell lymphoma (n = 10) are expressed relative to healthy controls (n = 9). Thick line represents median and X indicates the mean values.

FGL-2 mRNA and protein levels were studied in PBMC using RT-PCR or ELISA, respectively (Figure 2). In contrast to increased FGL-2 activity in PBMC from lymphoma patients no significant difference in FGL-2 mRNA was observed in the PBMC of normal controls (n = 13), patients with active B-cell lymphoma (n = 12) or lymphoma patients in remission (n = 7) (Figure 2A). Mean±SEM and median values are: for controls −100%±31% and 59%, respectively; for active lymphoma patients −77%±24% and 53%, respectively; for patients in remission −127%±85% and 61%, respectively (p = 0.48 for lymphoma at diagnosis versus controls; p = 0.74 for active lymphoma versus lymphoma in remission). Similarly, no significant difference in FGL-2 protein levels in the PBMC was observed between normal controls (n = 9) and patients with active lymphoma (n = 10) (Figure 2B). Mean±SEM and median values for controls: 100%±32% and 59%, respectively; for active lymphoma patients: 99%±30% and 54%, respectively, p = 0.986.

## Discussion

Up to now, increased FGL-2 expression was reported within tumor cells [15]. No data exist regarding FGL-2 activity or expression in PBMC of normal individuals or patients with malignancy.

In this work we measured FGL-2 prothrombinase activity in the PBMC of patients with active lymphoma or during remission, to determine whether it could serve as a potential marker for follow up of this malignancy. Indeed, prothrombinase activity of FGL-2 was found to be 3-fold higher than that of healthy controls, with sensitivity of 73.6% and specificity of 80.7% at a cutoff of 150% FGL-2 activity. The lack of an increase in FGL-2 prothrombinase activity in situation where extensive amount of thrombin is required, such as tissue injury during coronary surgery, as has been found in this work, implied that an increase activity observed in active lymphoma patient is associated with malignant process. Moreover, the observation that a significant decrease in FGL-2 activity was measured during remission of lymphoma may suggest that monitoring of FGL-2 activity may indicate the response to treatment. Taken together, our results suggest that FGL-2 prothrombinase activity is increased in active lymphoma patients and can be used as a potential marker for the recurrence of lymphoma during remission.

To date, the diagnosis of B-cell lymphoma is largely based on the pathologic workup of patients with suspicious clinical presentation. There is a deficiency of simple, non-invasive biomarkers that may assist in diagnosis and follow up of patients with lymphoma. The role of LDH as a biomarker in lymphoma has previously been explored. Published studies have demonstrated a rather low sensitivity for indolent and aggressive NHL (up to 38% and 60%, respectively) [23]. Recently, the sensitivity of LDH in detecting relapse of patients with DLBCL in complete remission was shown to be 69% albeit with a very low positive predictive value of 14% [24]. Various other hematologic, morphologic and recently described molecular and genetic markers have been studied, some of which were shown to correlate with disease biology and outcome although, the sensitivity and specificity of these biomarkers for diagnosis and follow-up are not well established [25]. In contrast to the increase in FGL-2 activity in PBMC of lymphoma patients, no increase in either mRNA or protein levels was observed. These findings may imply that the increase in FGL-2 activity measured in PBMC stems from post- translational regulation that governs final activity. While the mechanism is yet to be determined, it is already established that a linear correlation between RNA and protein expression profile is significant in a third of human genes [26].

It is intriguing to assume that increased activity of FGL-2 may have a role in lymphoma. It is possible that FGL-2 plays a role in this malignancy either directly or via thrombin generation. It has previously been reported that FGL-2 was detected in tumor tissues being upregulated in cancer cells and interstitial inflammatory cells, whereas the normal tissue surrounding the tumor did not display overexpression of FGL-2 [15]. According to Su et al., the observed pro-tumorigenic activity is not a direct effect of FGL-2 but rather a result of FGL-2 prothrombinase activity leading to the generation of thrombin with subsequent thrombin-induced tumorigenesis [15]. However, the reason for the upregulation of FGL-2 in tumor cells is not clear. As yet, all the publications on FGL-2 protein/expression have reported its upregulation only in malignant cells. To the best of our knowledge, this is a first report on the upregulation of FGL-2 prothrombinase activity in non-malignant PBMC of lymphoma patients. If our findings are confirmed by other studies, the assay of FGL-2 activity in PBMC could be used as a marker for follow up of B-cell lymphoma.

## References

[1] Blom JW, Osanto S, Rosendaal FR (2006) High risk of venous thrombosis in patients with pancreatic cancer: a cohort study of 202 patients. Eur J Cancer 42: 410–414. 16321518 10.1016/j.ejca.2005.09.013

[2] Trousseau A (1865) In Clinique Medicale de l'Hotel-dieu de Paris. Phlegmasia Alba Dolens. Paris: JB Balliere et Fils: 654–715.

[3] Blom JW, Doggen CJ, Osanto S, Rosendaal FR (2005) Malignancies, prothrombotic mutations, and the risk of venous thrombosis. Jama 293: 715–722. 15701913 10.1001/jama.293.6.715

[4] Blom JW, Vanderschoot JP, Oostindier MJ, Osanto S, van der Meer FJ, et al (2006) Incidence of venous thrombosis in a large cohort of 66,329 cancer patients: results of a record linkage study. J Thromb Haemost 4: 529–535. 16460435 10.1111/j.1538-7836.2006.01804.x

[5] Sorensen HT, Mellemkjaer L, Olsen JH, Baron JA (2000) Prognosis of cancers associated with venous thromboembolism. N Engl J Med 343: 1846–1850. 11117976 10.1056/NEJM200012213432504

[6] Caruso V, Iacoviello L, Di Castelnuovo A, Storti S, Donati MB (2007) Venous thrombotic complications in adults undergoing induction treatment for acute lymphoblastic leukemia: results from a meta-analysis. J Thromb Haemost 5: 621–623. 17229043 10.1111/j.1538-7836.2007.02383.x

[7] Shankland KR, Armitage JO, Hancock BW (2012) Non-Hodgkin lymphoma. Lancet 380: 848–857. 22835603 10.1016/S0140-6736(12)60605-9

[8] Fisher SG, Fisher RI (2004) The epidemiology of non-Hodgkin's lymphoma. Oncogene 23: 6524–6534. 15322522 10.1038/sj.onc.1207843

[9] Smith A, Howell D, Patmore R, Jack A, Roman E (2011) Incidence of haematological malignancy by sub-type: a report from the Haematological Malignancy Research Network. Br J Cancer 105: 1684–1692. 22045184 10.1038/bjc.2011.450 PMC3242607

[10] A predictive model for aggressive non-Hodgkin's lymphoma. The International Non-Hodgkin's Lymphoma Prognostic Factors Project. N Engl J Med 329: 987–994. 8141877 10.1056/NEJM199309303291402

[11] Geisler CH, Kolstad A, Laurell A, Raty R, Jerkeman M, et al (2010) The Mantle Cell Lymphoma International Prognostic Index (MIPI) is superior to the International Prognostic Index (IPI) in predicting survival following intensive first-line immunochemotherapy and autologous stem cell transplantation (ASCT). Blood 115: 1530–1533. 20032504 10.1182/blood-2009-08-236570

[12] Solal-Celigny P (2006) Follicular Lymphoma International Prognostic Index. Curr Treat Options Oncol 7: 270–275. 16916487 10.1007/s11864-006-0036-3

[13] Cheson BD (2005) Individualizing therapy for the hematologic malignancies: the stuff of genes and dreams. J Clin Oncol 23: 6283–6284. 16155010 10.1200/JCO.2005.08.005

[14] Ning Q, Sun Y, Han M, Zhang L, Zhu C, et al (2005) Role of fibrinogen-like protein 2 prothrombinase/fibroleukin in experimental and human allograft rejection. J Immunol 174: 7403–7411. 15905589 10.4049/jimmunol.174.11.7403

[15] Su K, Chen F, Yan WM, Zeng QL, Xu L, et al (2008) Fibrinogen-like protein 2/fibroleukin prothrombinase contributes to tumor hypercoagulability via IL-2 and IFN-gamma. World J Gastroenterol 14: 5980–5989. 18932275 10.3748/wjg.14.5980 PMC2760190

[16] Chan CW, Chan MW, Liu M, Fung L, Cole EH, et al (2002) Kinetic analysis of a unique direct prothrombinase, fgl2, and identification of a serine residue critical for the prothrombinase activity. J Immunol 168: 5170–5177. 11994472 10.4049/jimmunol.168.10.5170

[17] Chan CW, Kay LS, Khadaroo RG, Chan MW, Lakatoo S, et al (2003) Soluble fibrinogen-like protein 2/fibroleukin exhibits immunosuppressive properties: suppressing T cell proliferation and inhibiting maturation of bone marrow-derived dendritic cells. J Immunol 170: 4036–4044. 12682232 10.4049/jimmunol.170.8.4036

[18] Marazzi S, Blum S, Hartmann R, Gundersen D, Schreyer M, et al (1998) Characterization of human fibroleukin, a fibrinogen-like protein secreted by T lymphocytes. J Immunol 161: 138–147. 9647217

[19] Ruegg C, Pytela R (1995) Sequence of a human transcript expressed in T-lymphocytes and encoding a fibrinogen-like protein. Gene 160: 257–262. 7642106 10.1016/0378-1119(95)00240-7

[20] Ghanekar A, Mendicino M, Liu H, He W, Liu M, et al (2004) Endothelial induction of fgl2 contributes to thrombosis during acute vascular xenograft rejection. J Immunol 172: 5693–5701. 15100314 10.4049/jimmunol.172.9.5693

[21] Hancock WW, Szaba FM, Berggren KN, Parent MA, Mullarky IK, et al (2004) Intact type 1 immunity and immune-associated coagulative responses in mice lacking IFN gamma-inducible fibrinogen-like protein 2. Proc Natl Acad Sci U S A 101: 3005–3010. 14976252 10.1073/pnas.0308369101 PMC365735

[22] Liu Y, Xu L, Zeng Q, Wang J, Wang M, et al (2012) Downregulation of FGL2/prothrombinase delays HCCLM6 xenograft tumour growth and decreases tumour angiogenesis. Liver Int 32: 1585–1595. 22925132 10.1111/j.1478-3231.2012.02865.x

[23] Huijgen HJ, Sanders GT, Koster RW, Vreeken J, Bossuyt PM (1997) The clinical value of lactate dehydrogenase in serum: a quantitative review. Eur J Clin Chem Clin Biochem 35: 569–579. 9298346

[24] El-Sharkawi D, Basu S, Ocampo C, Qian W, D'Sa S, et al (2012) Elevated lactate dehydrogenase levels detected during routine follow-up do not predict relapse in patients with diffuse large B-cell lymphoma who achieve complete remission after primary treatment with rituximab, cyclophosphamide, doxorubicin, vincristine and prednisone-like immunochemotherapy. Leuk Lymphoma 53: 1949–1952. 22462615 10.3109/10428194.2012.679360

[25] Martelli M, Ferreri AJ, Agostinelli C, Di Rocco A, Pfreundschuh M, et al (2013) Diffuse large B-cell lymphoma. Crit Rev Oncol Hematol 87: 146–171. 23375551 10.1016/j.critrevonc.2012.12.009

[26] Gry M, Rimini R, Stromberg S, Asplund A, Ponten F, et al (2009) Correlations between RNA and protein expression profiles in 23 human cell lines. BMC Genomics 10: 365. 19660143 10.1186/1471-2164-10-365 PMC2728742

